# Increased Circulating Levels of Galectin Proteins in Patients with Breast, Colon, and Lung Cancer

**DOI:** 10.3390/cancers13194819

**Published:** 2021-09-26

**Authors:** Bailey B. Blair, Avery T. Funkhouser, Jane L. Goodwin, Alexander M. Strigenz, Basil H. Chaballout, Julie C. Martin, Connie M. Arthur, Christopher Ronald Funk, W. Jeffery Edenfield, Anna V. Blenda

**Affiliations:** 1 Department of Biomedical Sciences, University of South Carolina School of Medicine Greenville, Greenville, SC 29605, USA; bergmanb@email.sc.edu (B.B.B.); averytf@email.sc.edu (A.T.F.); goodwij6@mailbox.sc.edu (J.L.G.); strigenz@email.sc.edu (A.M.S.); basilc@email.sc.edu (B.H.C.); 2 Prisma Health Cancer Institute, Greenville, SC 29605, USA; Julie.Martin@prismahealth.org (J.C.M.); Jeffery.Edenfield@prismahealth.org (W.J.E.); 3 Joint Program in Transfusion Medicine, Department of Pathology, Brigham and Women’s Hospital, Harvard Medical School, Boston, MA 02115, USA; cmarthur@bwh.harvard.edu; 4 Department of Hematology and Medical Oncology, Emory University School of Medicine, Atlanta, GA 30322, USA; ronnie.funk@emory.edu

**Keywords:** galectin, cancer, breast, lung, colon, ELISA, biorepository

## Abstract

**Simple Summary:**

Galectins are a family of lectins that function to recognize carbohydrate groups, such as those on glycosylated proteins. Throughout tumor development, changes in the glycosylation patterns of surface proteins occur, often varying as the disease progresses. We show increased circulating levels of certain galectins in a biorepository of samples from patients with breast, colon, and lung cancer. Patterns of overexpression in cancer relative to healthy controls were observed, with galectins-1 and -3 increased in breast, colon, and lung cancer, while the level of galectin-7 was increased in breast and squamous cell lung cancer, and the level of galectin-9 was increased in colon and lung cancer. No noticeable trends in galectin levels were observed between cancer stages. These data suggest that dysregulation of galectins occurs early in oncogenesis. We also identified histologic subsets in lung cancer for which further investigation of galectins may yield useful diagnostic biomarkers.

**Abstract:**

Galectins are proteins with high-affinity β-galactoside-binding sites that function in a variety of signaling pathways through interactions with glycoproteins. The known contributions of galectins-1, -3, -7, -8, and -9 to angiogenesis, metastasis, cell division, and evasion of immune destruction led us to investigate the circulating levels of these galectins in cancer patients. This study compares galectin concentrations by enzyme-linked immunosorbent assay (ELISA) from each stage of breast, lung, and colon cancer. Galectins-1 and -7, which share a prototype structure, were found to have statistically significant increases in breast and lung cancer. Of the tandem-repeat galectins, galectin-8 showed no statistically significant change in these cancer types, but galectin-9 was increased in colon and lung cancer. Galectin-3 is the only chimera-type galectin and was increased in all stages of breast, colon, and lung cancer. In conclusion, there were significant differences in the galectin levels in patients with these cancers compared with healthy controls, and galectin levels did not significantly change from stage to stage. These findings suggest that further research on the roles of galectins early in disease pathogenesis may lead to novel indications for galectin inhibitors.

## 1. Introduction

Galectins are a family of lectin proteins that have high-affinity β-galactoside-binding sites. The galectins identified in humans to date share conserved homologous carbohydrate-recognition domains (CRDs) but differ in tertiary structure [1]. There are three subgroups of galectins based on the number and configuration of CRDs. The prototype subgroup is characterized by one CRD and includes galectins-1, -2, and -7. Galectin-3 is the sole member of the chimeric subtype and is able to form pentamers that may contribute to antiapoptotic signaling [2]. The last subtype contains the tandem-repeat galectins-4, -8, -9, and -12, which have two CRDs. The variations in structure and additional isoforms obtained from mRNA splicing confer unique functions based on binding capabilities. Some of these physiological functions include immune system response and cell signaling for division, migration, apoptosis, and autophagy [3].

Cancer is a leading health concern and is the second highest cause of death in the United States. Three of the top cancers by incidence include breast, lung and bronchus, and colorectal cancer [4]. Galectins participate in a variety of signaling pathways relevant to these cancers. These cellular processes include apoptosis, angiogenesis, cell adhesion (e.g., during metastasis), and immune response to cancer [5]. Different galectins have been shown to have varying effects on oncogenesis. Galectins-1 and -3 have demonstrated pro-neoplastic effects on tumor growth, angiogenesis, evasion of apoptosis, metastasis, and suppressing T-cell activity in the tumor microenvironment [6,7,8]. Circulating galectin-3 specifically has been found to induce secretion of cytokines contributing to metastasis, including interleukin-6 (IL-6), by endothelial cells [9]. The effects of the galectins can differ, depending on the cancer type. For example, galectin-7 has been shown to promote metastasis in breast cancer, but it is tumor suppressive in gastric cancer [10,11]. The effects of galectin-8 are poorly understood, but it has been shown to possibly promote metastasis of colon cancer by enhancing malignant cell adhesion to endothelium [12]. Specifically, a study showed that galectin-8 (as well as galectins-2 and -4) contributes to the expression of IL-6, a known promotor of cell adhesion, in serum samples from patients with breast and colon cancer [13]. Alternatively, galectin-9 prevents access to endothelium and promotes activation of natural killer cells [14,15]. Thus, galectins are significant in their roles in cancer progression and metastasis both as stimulators and inhibitors.

Several studies have looked into galectin expression levels in neoplastic diseases using immunohistochemistry (IHC) and enzyme-linked immunosorbent assays (ELISAs) with varying goals. Some provide mechanistic explanations of the galectin’s roles in cancer, such as tumor cell adhesion to endothelium and promotion of angiogenesis and metastasis [15,16]. More clinically focused studies look into the ability to detect breast cancer with galectins, monitor therapy response and tumor progression, serve as markers for colorectal cancer, and further investigate galectins’ prognostic value [17,18,19,20]. For example, a study investigating circulating galectins as colorectal cancer markers found that galectin-1 was significantly increased in patients with colorectal cancer compared with healthy controls. After surgical resection of the cancer, galectin-1 levels decreased substantially, suggesting the use of galectin-1 as a potentially more reliable measure of disease management [19]. However, many galectins, such as galectin-8, have not been studied to the same extent as others. Thus far, there are no comprehensive clinical studies on galectins, as most studies focus on one or two galectins in a specific disease type [21].

It has been noted that the use of different methods among different studies has made comparisons of findings difficult [21]. Thus, a singular, wider scope analysis is needed. This study reports the average levels of five circulating galectins in patient samples organized by stage in three types of cancer (breast, colon, and lung), which is a novel approach to comparing galectin data. This method allows for the creation of a comprehensive galectin profile in cancer patients. Galectins-1, -3, and -9 were selected due to their known roles in cancer and provide representation of each galectin subtype. Galectins-7 and -8 were also included due to their understudied status.

Currently, work is underway to use galectins as biomarkers and as targets for clinical therapy. Galectins have been investigated as markers for heart failure, atrial fibrillation, endometriosis, and various cancers [19,22,23,24,25,26,27,28,29,30,31,32]. Research and clinical trials are also considering galectins as potential therapeutic targets, where galectin-1 and -3 inhibitors are specifically being investigated [33,34,35]. Additionally, the galectin profiles of the patients generated from this study will improve the characterization of their disease state and allow for potential identification of patients who would benefit from participation in clinical trials of galectin inhibitors.

## 2. Materials and Methods

### 2.1. Cancer Patient Samples

Patient samples were obtained from the Prisma Health Cancer Institute’s biorepository (PHCI). Selection of cancer types for analysis was based on the overall cancer rank in incidence and specimen availability in a community biorepository. Patients were consented at the time of tissue procurement.

Forty serum samples were obtained from patients with breast cancer (95% ductal, 5% lobular). Seventeen serum samples and 20 plasma samples were obtained from patients with colon cancer (92% adenocarcinoma, 8% unknown). Forty serum samples were obtained from patients with lung cancer (62.5% adenocarcinoma, 32.5% squamous cell carcinoma, 5% large cell carcinoma). Ten samples were acquired for each stage of each cancer, except for stage III of colon cancer, which had 7 samples. A power analysis was performed, and the sample sizes were determined to be adequate for the scope and scale of the study.

Most of the specimens (78.07%) came from treatment naïve patients newly diagnosed with cancer. A small number (4.39%) of specimens came from recurrent cancers, and those patients had not recently received treatment. Another group of samples (16.67%) came from metastatic cancers, and in most cases, the patients had recently had or were on chemotherapy. One specimen, comprising 0.88% of the data set, had unknown treatment status (Tables S1–S3).

### 2.2. Healthy Controls

The values for healthy control mean and standard deviation (SD) of the serum levels of galectins-1, -3, and -9 were obtained by R&D Systems (Minneapolis, MN, USA) from 36 healthy volunteers. Additionally, 27 serum samples from healthy persons were obtained from BioChemed Services (Winchester, VA, USA).

### 2.3. ELISA Analysis

ELISA kits for galectins-1, -3, and -9 (R&D Systems, Minneapolis, MN, USA) and galectins-7 and -8 (Invitrogen, Carlsbad, CA, USA) were used to conduct analysis according to manufacturer protocol. Microplate readings of optical density (OD) were wavelength-corrected (450–570 nm readings) and used to obtain optical density values. The average OD of each sample was calculated and blank-corrected. A four-parameter logistic curve was used to calculate the galectin concentration from the OD reading, which was multiplied by the dilution factor to arrive at the circulating galectin concentration (Tables S4 and S5).

### 2.4. Data Analysis

Analysis of the galectin levels was performed using JMP^®^ by SAS Institute (Cary, NC, USA). The distributions of the circulating galectin values were determined to be non-normal, and thus nonparametric tests were used. The Wilcoxon signed rank test was used to compare any two groups of galectin values. The Steel test for multiple comparisons with a control was used to compare the mean galectin levels of cancer patient stages with the healthy control group (Tables S4 and S5)

Each galectin level in each cancer population was compared with the healthy control values. The galectin levels in each of the cancer stages were compared with healthy controls.

Analysis of breast and colon cancer by histological subtype was not performed due to the low number of certain subtypes of cancer samples. Analysis of lung cancer galectin levels was performed by histological subtypes including squamous cell carcinoma and adenocarcinoma.

## 3. Results

### 3.1. Galectins Are Elevated in Sera of Patients with Breast, Lung, and Colon Cancer

To assess for differences in the levels of circulating galectins in patients with breast, lung, and colon cancer relative to healthy controls, we obtained samples from the Prisma Health Cancer Institute’s biorepository. Patient characteristics are shown in Tables S1–S3 for breast (*n* = 40), colon (*n* = 37), and lung cancer (*n* = 40) respectively. To begin, ELISA was performed on cryopreserved serum (breast and lung cancers) and serum and plasma (colon cancer) on these cohorts of patients. Data were first analyzed by comparing disease samples with healthy donor samples to investigate whether differences in the circulating galectin levels were detectable relative to healthy controls (as obtained from the manufacturer as described in the methods).

**Figure 1:**
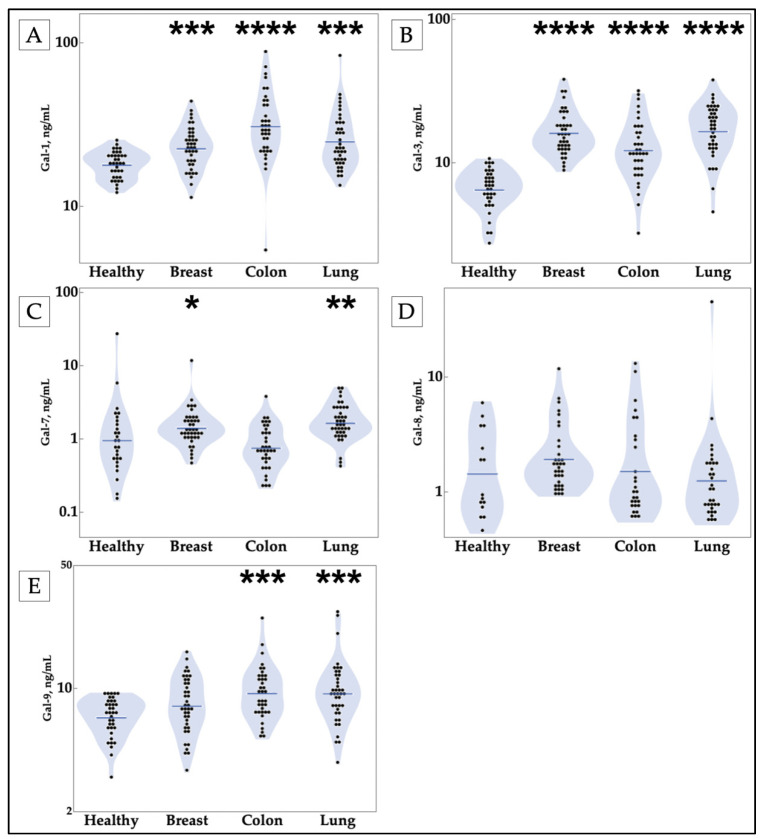
Circulating concentrations of galectins-1, -3, -7, and -9 are increased in breast, colon, and lung cancer patients compared with healthy controls. Cancer galectin levels were determined by ELISA of patient samples. (**A**,**B**) Galectins-1 and -3 are increased in all three cancer types, (**C**) galectin-7 is increased in breast and lung cancer patient samples, (**D**) galectin-8 shows no statistically significant differences from the healthy control, and (**E**) galectin-9 is found to be statistically significantly increased in colon and lung cancer. Stars indicate the *p*-values of nonparametric comparisons with control using the Steel method (* *p* ≤ 0.05, ** *p* ≤ 0.01, *** *p* ≤ 0.001, **** *p* ≤ 0.0001). Gal = galectin.

Through this initial screening, the galectins of each structural type are elevated. Prototypical galectins-1 and -7, which are dimers that are not linked by a linker sequence, were both elevated in breast and lung cancer (Figure 1A,C), whereas only galectin-1 was elevated in colon cancer. The chimeric galectin-3, which consists of monomers that can be arranged in different spatial configurations, was also elevated in all cancers tested (Figure 1B). Lastly, the tandem-repeat galectins, which consist of two similar domains linked by a linking sequence, galectin-8 and -9, showed mixed results. No significant changes in galectin-8 were observed in cancer relative to healthy controls (Figure 1D). In contrast, galectin-9 was elevated in colon and lung cancer patients (Figure 1E). This broad screening analysis informed additional subanalyses where galectin levels were assessed in samples from varying stages and histologic subtypes.

### 3.2. Breast Cancer

**Figure 2:**
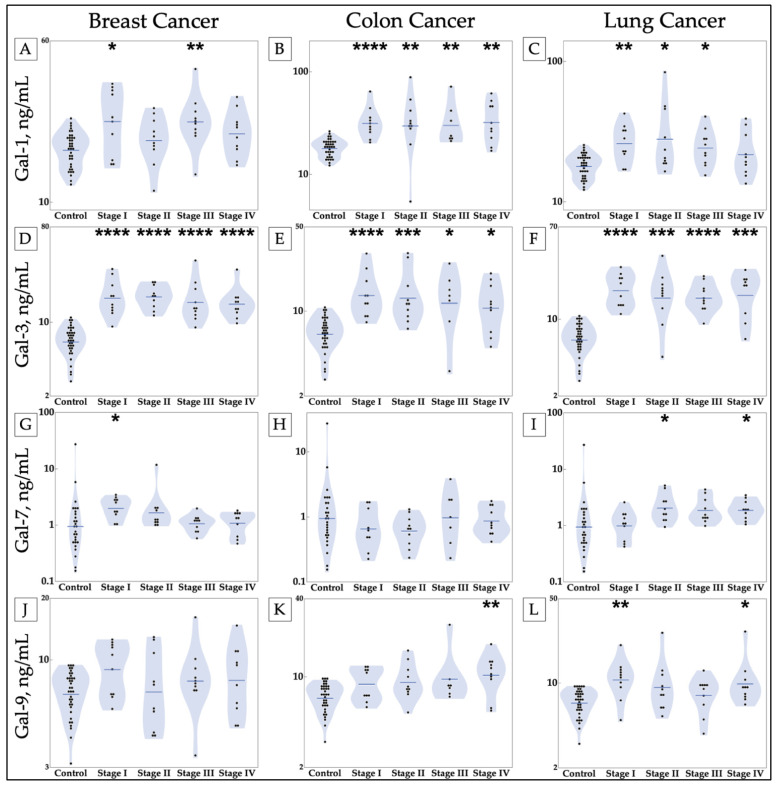
Elevations in circulating levels of galectins-1, -3, -7, and -9 occur in breast, colon, and lung cancer patients compared with healthy controls. Cancer galectin levels were determined by ELISA of patient samples. (**A**) A statistically significant difference in galectin-1 is shown between stages I and III of breast cancer and healthy controls; (**B**) galectin-1 is shown to be increased in all stages of colon cancer compared with healthy controls; (**C**) a statistically significant difference in galectin-1 is shown in stages I, II, and III of lung cancer. (**D**–**F**) Galectin-3 is observed to be elevated in all stages of all cancers, (**G**–**I**) a statistically significant increase in serum galectin-7 is observed in stage I of breast cancer and stages II and IV of lung cancer, and (**J**–**L**) galectin-9 is shown to be increased in stage IV of colon cancer and stages I and IV of lung cancer as compared with healthy controls. Stars indicate the *p*-values of nonparametric comparisons with control using the Steel method (* *p* ≤ 0.05, ** *p* ≤ 0.01, *** *p* ≤ 0.001, **** *p* ≤ 0.0001). Gal = galectin.

Given the finding of significant increase in galectins in the patient serum samples of breast cancer as compared with healthy controls, the data were then separated by stage for additional analysis. There was a statistically significant increase in the mean concentrations of galectin-1 in stages I and III (*p* = 0.0456, *p* = 0.0025, respectively), but none for stages II and IV (Figure 2A). An increase in the concentration of galectin-3 was seen in all stages with a significance of less than 0.0001 (Figure 2D). Galectin-7 was only elevated in stage I (*p* = 0.0206) and not in later stages of breast cancer (Figure 2G).

Next, the data were organized by other known patient characteristics, including sex, race, and smoking status, and tumor characteristics including stage and histological subtype, as seen in Table 1. No correlation was detected between demographic features and tumor characteristics and the concentrations of galectins-1, -3, -7, -8, and -9 in serum samples of patients diagnosed with breast cancer.

### 3.3. Colon Cancer

The serum and plasma samples of patients with identified colon cancer were also separated by stage for additional analysis after finding that galectins-1, -3, and -9 were increased in the serum and plasma of colon cancer patients included in this study (*p* < 0.0001, *p* < 0.0001, and *p* = 0.0005, respectively), as seen in Figure 1A,B,E. Galectin-1 appears to be consistently elevated throughout the progression of colon cancer with increases observed in stages I–IV (*p* < 0.0001, *p* < 0.0022, *p* < 0.0026, *p* < 0.0014, respectively) (Figure 2B). Galectin-3 is also significantly elevated in all stages with statistically significant *p*-values (*p* < 0.0001, *p* < 0.0002, *p* < 0.0235, and *p* < 0.0152) (Figure 2E). In contrast, galectin-9 was found to be increased in stage IV with a *p*-value of 0.0044, but had no reportable changes for stages I, II, and III (Figure 2K).

Statistical analysis was performed with patient characteristics as described above, and higher levels of galectin-7 were seen in patients who reported their race as white. Levels of galectin-7 were also higher in patients who had a history of smoking. Galectin-8 was observed to be increased more in male patients with colon cancer compared to female patients with colon cancer (Table 1).

### 3.4. Lung Cancer

Galectin-1, -3, -7, and -9 were found to be significantly elevated in lung cancer patients (*p* = 0.0002, *p* < 0.0001, *p* = 0.0071, and *p* = 0.0002, respectively) in Figure 1A–C,E. Thus, the data for these galectins were further studied with stage separation. Galectin-1 is elevated in stages I, II, and III with *p*-values of 0.0036, 0.0364, and 0.0179, but not significantly changed in stage IV as compared with healthy controls (Figure 2C). Galectin-3 is increased in all four stages compared with healthy controls (*p* < 0.0001, *p* = 0.0005, *p* < 0.0001, *p* = 0.0004, respectively), as seen in Figure 2F. Galectin-7 is elevated in stages II and IV with *p*-values of 0.0341 and 0.0341, with no significant increase or decrease in serum levels for stages I and III (Figure 2I). There was no change in the serum levels of galectin-9 in the serum of the studied patients with stage II or III lung cancer, but galectin-9 was found to be elevated in stages I and IV (*p* = 0.0030, *p* = 0.0179, respectively) (Figure 2L).

**Figure 3:**
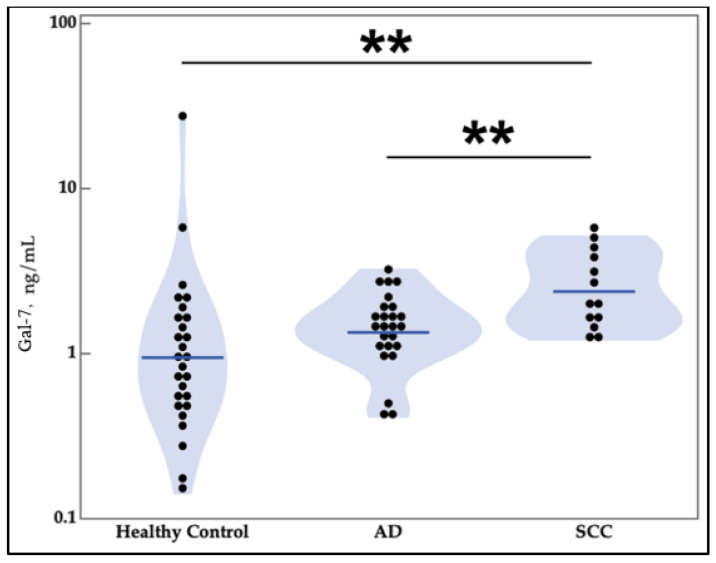
Lung squamous cell carcinoma patients have increased serum levels of galectin-7 relative to lung adenocarcinoma patients. AD is adenocarcinoma. SCC is squamous cell carcinoma. Cancer galectin levels were determined by ELISA of patient serum samples. SCC and healthy control comparison was made using nonparametric comparison with control using the Steel method. SCC and AD comparison was made using the Wilcoxon test (** *p* ≤ 0.01). Gal = galectin.

Regarding the statistical analysis for correlations based on patient characteristics, an increase in galectins-3 and -9 was observed in female patients compared with male patients (Table 1). Galectin-9 was increased in patients who had never smoked compared with current smokers. Galectin-7 was statistically significantly lower in the first stage of lung cancer than the rest of the stages. There was an intriguing finding that galectin-7 was elevated in serum samples from squamous cell lung cancer patients relative to healthy controls and adenocarcinoma lung cancer patients (Figure 3).

## 4. Discussion

### 4.1. Significance of the Study

Peripheral blood biomarkers are needed to facilitate early diagnosis of malignancy. Our study shows profiles of galectin expression in patients with breast, colon, and lung cancer and identifies galectin–disease combinations that warrant further investigation. A review of published literature showed inconsistent alterations in galectin expression in tumor versus normal tissue and noted that most studies use fewer galectins and cancer types compared with this study [21]. The findings of this study compared with the review article can be seen in Table 2. They also highlight the potential areas for future study, such as elevated levels of galectin-9 in lung cancer samples, which had not been identified previously.

Our study builds upon published literature and provides data on circulating levels of five galectins for three cancer types, utilizing a consistent method of ELISA analysis, thus enabling comparison of galectin levels across tumor types. Other studies using ELISA for the determination of serum galectin values are varied in their comparison of cancer patients versus healthy individuals; however, they tend to agree that most galectin levels increase in patients with neoplastic disease [15,16,17]. The opposing findings in the literature are potentially due to differences in methodology, sensitivity of the ELISA kit used, sample preparation, and cancer (sub)types. This demonstrates a need for more standardization in the measurement of galectin expression levels.

We demonstrate elevated levels of circulating galectins in three different cancers, beginning with stage I disease, suggesting that galectins may have a role as a diagnostic biomarker (Figure 2). Galectin-3 was elevated in breast, colon, and lung cancer in our data (Figure 2); however, galectin-3 is also implicated in a variety of diseases, including heart failure with reduced ejection fraction, chronic obstructive pulmonary disease, diabetes mellitus, and chronic kidney disease [36]. Our analysis is limited in that patient comorbidities were not available for statistical analysis. Therefore, circulating values of galectin-3 may represent a sensitive but nonspecific test in diagnosing breast, colon, or lung cancer. Further testing with larger sample groups may lead to the development of galectin “fingerprints”, unique combinations of circulating galectin levels, for different cancers. High-throughput ELISA could potentially make this a practical test. Other studies are also finding value in using combinations of galectins for diagnostic and prognostic purposes [20,37].

### 4.2. Proposed Mechanisms

Additionally, our study shows that galectin-7 is elevated in lung cancer patients with a squamous cell tumor histology. Galectin-7 is highly expressed in epithelial tissues, especially stratified squamous epithelium, which corroborates this finding [38]. Galectin-7 was found to play a role in the pathogenesis of psoriasis, an inflammatory process affecting stratified squamous cells of the skin by elevating levels of IL-6 and IL-8 [39]. The elevated serum levels of galectin-7 in patients with tumors of squamous cell origin help to explain the origin of galectins in the serum. It is possible that the uncontrolled growth of these galectin-7-expressing cells causes the increase in galectin-7 in the serum as a byproduct of having more galectin-7 production by the tumor. Further testing is needed to confirm whether galectin-7 levels could be a differentiating factor for identifying lung cancer tumor types.

Galectins-1 and -3 were shown to play a salient role in the promotion or inhibition of the putative hallmarks of cancer [7]. Galectin-1 can bind to HRAS, and galectin-3 can associate with basal bodies and centrosomes as well as a mitotic regulator known as nuclear mitotic apparatus protein (NuMa) [40,41,42]. The process of programmed cell death, apoptosis, is also impacted by galectin-3 as it is involved in the β-catenin/Wnt pathway and regulation with Bcl-2 [43,44].

Alternatively, increased or decreased levels of other galectins may be due to the body’s response to cancer. Galectin-9 is heavily involved in immune function and is a negative indicator of metastatic ability in breast cancer [14,45,46,47]. Thus, higher circulating levels of this galectin may indicate the body’s increased immune response to the tumor. However, galectin-9 has also been shown to bind to T cell immunoglobulin mucin 3 (TIM-3), which dampens the effector T cell response by initiating apoptosis. Monocytes and dendritic cells also express TIM-3, and it has been suggested that the binding of galectin-9 can block maturation and cytokine production [48]. Whether the elevations in galectins are due to tumor-intrinsic mechanisms or due to the immune response to cancer, galectins may prove to be a useful biomarker for early detection of common malignancies.

### 4.3. Confounding Variables

Our study used samples that varied in specimen age (mean, 5.5 years; SD, 1.4 years). A previous study suggested that galectin-9 in particular was relatively unstable in frozen serum samples when stored for more than 6 months [29]. We ran bivariate analysis that confirmed no correlation between our specimen age and the levels of galectins-1, -3, -7, -8, and -9 (Spearman *p*: −0.05, −0.02, −0.09, −0.05, −0.006, respectively). Similarly, we found no correlation between galectin levels and the age of the patients at sample collection.

Half of our colon cancer samples came from plasma and not serum based on their availability in a community biorepository. There were no significant differences found between the galectin levels in the colon serum samples and the plasma samples through statistical analysis by the Wilcoxon signed rank test (galectin-1, 0.3686; galectin-3, 0.2660; galectin-7, 0.1475; galectin-8, 0.8440; galectin-9, 0.6808). Thus, these samples were analyzed and compared together without any distinction being made.

It is a valid concern to correct for the treatments of the cancer patients as these could have an impact on the galectin levels. Galectins were shown to complicate the treatment of cancer patients by conferring resistance to therapies, including chemotherapy, immunotherapy, radiation, targeted therapies, and antiangiogenic therapies [49]. While specific treatment information was not collected on these patients, we determined the patients who had received cancer treatment and those who had not undergone treatment or had not received treatment recently. We found that the galectin levels of the treated patients versus those of the nontreated and not recently treated patients had no observable difference. Furthermore, upon exclusion of the 19 (16.67%) chemotherapy treated patients from the analysis, the outcomes presented in this paper did not change. Lastly, the data we present are correlative, and associations will have to be verified by mechanistic and clinical cohort studies.

### 4.4. Limitations

Our study is not without limitations. A sample size of approximately 40 samples per cancer potentially limits the generalizability of the study. Additionally, this study only evaluates information about a patient sample at one point in time. A cohort study following patients through the cancer progression and gathering samples at each stage may show more patterns of galectin expression throughout disease progression. Next, we were unable to identify patient comorbidities that could confound results due to limitations of our IRB protocol. Healthy control samples were not taken from the same biorepository as the patient samples and were not frozen for the same duration. The control means and SD for galectins-1, -3, and -9 were provided by R&D Systems as part of the ELISA kits. The use of a cohort study that follows patients from early on (stage 0) would allow for greater homogeneity in the sample sources.

It should be noted that the levels of circulating galectins could be due to differing expression levels in the neoplasm, which can upregulate specific galectins to promote angiogenesis and metastasis, or the levels of galectins could be due to healthy tissue changing its expression of galectins in response to the neoplastic tissue. Other studies suggest that the source of galectins can be tumor cells, surrounding stromal cells, or immune cells [13,19,50]. Considering that inflammation contributes to pathogenesis, this is a potential area of future study to determine the tissues that contribute the most to the elevation of circulating galectin levels seen in these cancers at early stages.

## 5. Conclusions

The majority of previous studies on galectins examined one or two types of galectins at a time [21]. This pilot study provides a more comprehensive look into multiple galectins in multiple cancers. We found that galectin-7 is significantly elevated in lung cancer patients with a squamous cell histology. Additionally, our study provided galectin profiles that are being incorporated into the Prisma Health Cancer Institute’s biorepository database. A similar approach could be adopted by other cancer biorepositories. These galectin profiles could contribute to future selection of medications for these patients, including galectin-based immunotherapies, which are currently under development [48,51]. In addition to being useful biomarkers for disease, galectins are potential targets for cancer therapy due to their well-documented role in cancer progression [33,34,35]. While the results are promising, additional information is needed to solidify and expand the role of galectin profiles in oncology.

## Figures and Tables

**Table 1:** Correlation analysis of galectin levels against cancer patient and tumor characteristics. Significant Wilcoxon *p*-values of one-way comparisons.

	Colon	Lung
Sex		
Gal-3 *		Female > Male 0.0472
Gal-8	Male > Female 0.0477	
Gal-9		Female > Male 0.0249
Race		
Gal-7	White > African-American 0.0143	
Smoking History		
Gal-7	Previous > Current 0.0436	
Gal-9		Never > Current 0.0436
Stage		
Gal-7		I < II, III, IV 0.0113, 0.0257, 0.0376
Histology		
Gal-7		SCC > AD 0.0056

* Gal = galectin, SCC = Squamous Cell Carcinoma, AD = Adenocarcinoma.

**Table 2:** Comparison of galectin level changes in cancer patients.

			Literature [21]	
Cancer	Galectin	Results	Serum	Tissue
	1	↑	↓	↑
	3	↑	↑	↑, ↓
Breast	7	↑	-	↑
	8	=	↑	↑
	9	=	=	-
	1	↑	↑, =, ↓	↑
	3	↑	↑	↑, ↓, =
Colon	7	=	=	↑
	8	=	↑	↓
	9	↑	↑	-
	1	↑	↑	↑
	3	↑	-	↑
Lung	7	↑	-	=
	8	=	-	=, ↑
	9	↑	-	-

=, expression unaltered; ↑, expression increased; ↓, expression decreased in cancerous samples vs. normal/benign samples; -, no data available. Differences are highlighted in yellow. Multiple outcomes are highlighted in gray.

## Data Availability

Data described in this paper are contained in the manuscript.

## Supplementary Materials

The following are available online at www.mdpi.com/article/10.3390/cancers13194819/s1, Table S1: Breast cancer patient demographics, Table S2: Lung cancer patient demographics, Table S3: Colon cancer patient demographics, Table S4: Galectin concentration values for patient groups in Figure 1, Table S5: Galectin concentration values for patient groups in Figure 2.

## References

[1] Barondes S.H., Cooper D.N.W., Gitt M.A., Leffler H. (1994). Structure and function of a large family of animal lectins. J. Biol. Chem..

[2] Ahmad N., Gabius H.-J., Andre S., Kaltner H., Sabesan S., Roy R., Liu B., Macaluso F., Brewer C.F. (2004). Galectin-3 precipitates as a pentamer with synthetic multivalent carbohydrates and forms heterogeneous cross-linked complexes. J. Biol. Chem..

[3] Johannes L., Jacob R., Leffler H. (2018). Galectins at a glance. J. Cell Sci..

[4] Siegel R.L., Miller K.D., Fuchs H.E., Jemal A. (2021). Cancer Statistics, 2021. CA A Cancer J. Clin..

[5] Liu F.-T., Rabinovich G.A. (2005). Galectins as modulators of tumour progression. Nat. Rev. Cancer.

[6] Ebrahim A.H., Alalawi Z., Mirandola L., Rakhshanda R., Dahlbeck S., Nguyen D., Jenkins M., Grizzi F., Cobos E., Figueroa J.A. (2014). Galectins in cancer: Carcinogenesis, diagnosis and therapy. Ann. Transl. Med..

[7] Girotti M.R., Salatino M., Dalotto-Moreno T., Rabinovich G.A. (2020). Sweetening the hallmarks of cancer: Galectins as multifunctional mediators of tumor progression. J. Exp. Med..

[8] Markowska A.I., Liu F.T., Panjwani N. (2010). Galectin-3 is an important mediator of VEGF- and bFGF-mediated angiogenic response. J. Exp. Med..

[9] Chen C., Duckworth C.A., Zhao Q., Pritchard D.M., Rhodes J.M., Yu L.G. (2013). Increased circulation of galectin-3 in cancer induces secretion of metastasis-promoting cytokines from blood vascular endothelium. Clin. Cancer Res..

[10] Campion C.G., Labrie M., Lavoie G., St-Pierre Y. (2013). Expression of Galectin-7 Is Induced in Breast Cancer Cells by Mutant p53. PLoS ONE.

[11] Kim S.J., Hwang J.A., Ro J.Y., Lee Y.S., Chun K.H. (2013). Galectin-7 is epigenetically-regulated tumor suppressor in gastric cancer. Oncotarget.

[12] Barrow H., Rhodes J.M., Yu L.-G. (2011). The role of galectins in colorectal cancer progression. Int. J. Cancer.

[13] Chen C., Duckworth C.A., Fu B., Pritchard D.M., Rhodes J.M., Yu L.G. (2014). Circulating galectins-2,-4 and-8 in cancer patients make important contributions to the increased circulation of several cytokines and chemokines that promote angiogenesis and metastasis. Br. J. Cancer.

[14] Hirashima M., Nagahara K., Oomizu S., Katoh S., Nishi N., Takeshita K., Niki T., Tominaga A., Yamauchi A., Nabumoto A. (2008). Galectin-9 suppresses tumor metastasis by blocking adhesion to endothelium and extracellular matrices. Glycobiology.

[15] Irie A., Yamauchi A., Kontani K., Kihara M., Liu D., Shirato Y., Seki M., Nishi N., Nakamura T., Yokomise H. (2005). Galectin-9 as a prognostic factor with antimetastatic potential in breast cancer. Clin. Cancer Res..

[16] Barrow H., Guo X., Wandall H.H., Pedersen J.W., Fu B., Zhao Q., Chen C., Rhodes J.M., Yu L.G. (2011). Serum galectin-2, -4, and -8 are greatly increased in colon and breast cancer patients and promote cancer cell adhesion to blood vascular endothelium. Clin. Cancer Res..

[17] Topcu T.O., Kavgaci H., Gunaldi M., Kocoglu H., Akyol M., Mentese A., Yaman S.O., Orem A., Ozdemir F., Aydin F. (2018). The clinical importance of serum galectin-3 levels in breast cancer patients with and without metastasis. J. Cancer Res. Ther..

[18] Saussez S., Lorfevre F., Lequeux T., Laurent G., Chantrain G., Vertongen F., Toubeau G., Decaestecker C., Kiss R. (2008). The determination of the levels of circulating galectin-1 and -3 in HNSCC patients could be used to monitor tumor progression and/or responses to therapy. Oral Oncol..

[19] Watanabe M., Takemasa I., Kaneko N., Yokoyama Y., Matsuo E.-I., Iwasa S., Mori M., Matsuura N., Monden M., Nishimura O. (2011). Clinical significance of circulating galectins as colorectal cancer markers. Oncol. Rep..

[20] Trebo A., Ditsch N., Kuhn C., Heidegger H.H., Zeder-Goess C., Kolben T., Czogalla B., Schmoeckel E., Mahner S., Jeschke U. (2020). High Galectin-7 and Low Galectin-8 Expression and the Combination of both are Negative Prognosticators for Breast Cancer Patients. Cancers.

[21] Thijssen V.L., Heusschen R., Caers J., Griffioen A.W. (2015). Galectin expression in cancer diagnosis and prognosis: A systematic review. Biochim. Biophys. Acta.

[22] Medvedeva E.A., Berezin I.I., Surkova E.A., Yaranov D.M., Shchukin Y.V. (2016). Galectin-3 in patients with chronic heart failure: Association with oxidative stress, inflammation, renal dyunction and prognosis. Minerva Cardioangiol..

[23] Than T.H., Swethadri G.K., Wong J., Ahmad T., Jamil D., Maganlal R.K., Hamdi M.M., Abdullah M.S. (2008). Expression of Galectin-3 and Galectin-7 in thyroid malignancy as potential diagnostic indicators. Singap. Med. J..

[24] Chiu C.G., Strugnell S.S., Griffith O.L., Jones S.J.M., Gown A.M., Walker B., Nabi I.R., Wiseman S.M. (2010). Diagnostic utility of galectin-3 in thyroid cancer. Am. J. Pathol..

[25] Sumana B.S., Shashidhar S., Shivarudrappa A.S. (2015). Galectin-3 immunohistochemical expression in thyroid neoplasms. J. Clin. Diagnostic Res..

[26] Zhu X., Ding M., Yu M.L., Feng M.X., Tan L.J., Zhao F.K. (2010). Identification of galectin-7 as a potential biomarker for esophageal squamous cell carcinoma by proteomic analysis. BMC Cancer.

[27] Yu X., Sun Y., Zhao Y., Zhang W., Yang Z., Gao Y., Cai H., Li Y., Wang Q., Bian B. (2015). Prognostic Value of Plasma Galectin-3 Levels in Patients with Coronary Heart Disease and Chronic Heart Failure. Int. Heart J..

[28] Takemoto Y., Ramirez R.J., Yokokawa M., Kaur K., Ponce-Balbuena D., Sinno M.C., Willis B.C., Ghanbari H., Ennis S.R., Guerrero-Serna G. (2016). Galectin-3 Regulates Atrial Fibrillation Remodeling and Predicts Catheter Ablation Outcomes. JACC Basic to Transl. Sci..

[29] Clementy N., Benhenda N., Piver E., Pierre B., Bernard A., Fauchier L., Pages J.C., Babuty D. (2016). Serum galectin-3 levels predict recurrences after ablation of atrial fibrillation. Sci. Rep..

[30] Wu X.Y., Li S.N., Wen S.N., Nie J.G., Deng W.N., Bai R., Liu N., Tang R.B., Zhang T., Du X. (2015). Plasma galectin-3 predicts clinical outcomes after catheter ablation in persistent atrial fibrillation patients without structural heart disease. Europace.

[31] Brubel R., Bokor A., Pohl A., Schilli G.K., Szereday L., Bacher-Szamuel R., Rigo J., Polgar B. (2017). Serum galectin-9 as a noninvasive biomarker for the detection of endometriosis and pelvic pain or infertility-related gynecologic disorders. Fertil. Steril..

[32] Kaneko N., Gotoh A., Okamura N., Matsuo E.I., Terao S., Watanabe M., Yamada Y., Hamami G., Nakamura T., Ikekita M. (2013). Potential tumor markers of renal cell carcinoma: α-Enolase for postoperative follow up, and galectin-1 and galectin-3 for primary detection. Int. J. Urol..

[33] Mirandola L., Nguyen D.D., Rahman R.L., Grizzi F., Yuefei Y., Figueroa J.A., Jenkins M.R., Cobos E., Chiriva-Internati M. (2014). Anti-galectin-3 therapy: A new chance for multiple myeloma and ovarian cancer?. Int. Rev. Immunol..

[34] Blanchard H., Bum-Erdene K., Bohari M.H., Yu X. (2016). Galectin-1 inhibitors and their potential therapeutic applications: A patent review. Expert Opin. Ther. Pat..

[35] Blanchard H., Yu X., Collins P.M., Bum-Erdene K. (2014). Galectin-3 inhibitors: A patent review (2008–present). Expert Opin. Ther. Pat..

[36] Sciacchitano S., Lavra L., Morgante A., Ulivieri A., Magi F., De Francesco G.P., Bellotti C., Salehi L.B., Ricci A. (2018). Galectin-3: One Molecule for an Alphabet of Diseases, from A to Z. Int. J. Mol. Sci..

[37] Carlsson M.C., Balog C.I.A., Kilsgård O., Hellmark T., Bakoush O., Segelmark M., Fernö M., Olsson H., Malmström J., Wuhrer M. (2012). Different fractions of human serum glycoproteins bind galectin-1 or galectin-8, and their ratio may provide a refined biomarker for pathophysiological conditions in cancer and inflammatory disease. Biochim. Biophys. Acta-Gen. Subj..

[38] Magnaldo T., Fowlis D., Darmon M. (1998). Galectin-7, a marker of all types of stratified epithelia. Differentiation.

[39] Chen H.-L., Lo C.-H., Huang C.-C., Lu M.-P., Hu P.-Y., Chen C.-S., Chueh D.-Y., Chen P., Lin T.-N., Lo Y.-H. (2021). Galectin-7 downregulation in lesional keratinocytes contributes to enhanced IL-17A signaling and skin pathology in psoriasis. J. Clin. Investig..

[40] Paz A., Haklai R., Elad-Sfadia G., Ballan E., Kloog Y. (2001). Galectin-1 binds oncogenic H-Ras to mediate Ras membrane anchorage and cell transformation. Oncogene.

[41] Koch A., Poirier F., Jacob R., Delacour D. (2010). Galectin-3, a novel centrosome-associated protein, required for epithelial morphogenesis. Mol. Biol. Cell.

[42] Magescas J., Sengmanivong L., Viau A., Mayeux A., Dang T., Burtin M., Nilsson U.J., Leffler H., Poirier F., Terzi F. (2017). Spindle pole cohesion requires glycosylation-mediated localization of NuMA. Sci. Rep..

[43] Shimura T., Takenaka Y., Fukumori T., Tsutsumi S., Okada K., Hogan V., Kikuchi A., Kuwano H., Raz A. (2005). Implication of galectin-3 in Wnt signaling. Cancer Res..

[44] Harazono Y., Nakajima K., Raz A. (2014). Why anti-Bcl-2 clinical trials fail: A solution. Cancer Metastasis Rev..

[45] Madireddi S., Eun S.Y., Lee S.W., Nemčovičová I., Mehta A.K., Zajonc D.M., Nishi N., Niki T., Hirashima M., Croft M. (2014). Galectin-9 controls the therapeutic activity of 4-1BB-targeting antibodies. J. Exp. Med..

[46] Meggyes M., Miko E., Polgar B., Bogar B., Farkas B., Illes Z., Szereday L. (2014). Peripheral blood TIM-3 Positive NK and CD8+ T cells throughout pregnancy: TIM-3/Galectin-9 interaction and its possible role during pregnancy. PLoS ONE.

[47] Nobumoto A., Oomizu S., Arikawa T., Katoh S., Nagahara K., Miyake M., Nishi N., Takeshita K., Niki T., Yamauchi A. (2009). Galectin-9 expands unique macrophages exhibiting plasmacytoid dendritic cell-like phenotypes that activate NK cells in tumor-bearing mice. Clin. Immunol..

[48] Yang R., Sun L., Li C.F., Wang Y.H., Yao J., Li H., Yan M., Chang W.C., Hsu J.M., Cha J.H. (2021). Galectin-9 interacts with PD-1 and TIM-3 to regulate T cell death and is a target for cancer immunotherapy. Nat. Commun..

[49] Navarro P., Martínez-Bosch N., Blidner A.G., Rabinovich G.A. (2020). Impact of Galectins in Resistance to Anticancer Therapies. Clin. Cancer Res..

[50] Sanjuan X., Fernandez P.L., Castells A., Castronovo V., Van den Brule F., Liu F.T., Cardesa A., Campo E. (1997). Differential expression of galectin 3 and galectin 1 in colorectal cancer progression. Gastroenterology.

[51] Wdowiak K., Francuz T., Gallego-Colon E., Ruiz-Agamez N., Kubeczko M., Grochoła I., Wojnar J. (2018). Galectin Targeted Therapy in Oncology: Current Knowledge and Perspectives. Int. J. Mol. Sci..

