# Supplementary material for: Increased Circulating Levels of Galectin Proteins in Patients with Breast, Colon, and Lung Cancer
Source: Cancers (Basel). 2021 Sep 26;13(19):4819. doi: 10.3390/cancers13194819 (PMC8508020; doi:10.3390/cancers13194819)
Supplement: Supplementary file 1 [file cancers-13-04819-s001.zip › cancers-1350701-supplementary.pdf]

## Supplemental Materials

**Table S1.** Breast cancer patient demographics

| Patient Characteristics (%) | Entire Population (n=40) | Stage I (n=10) | Stage II (n=10) | Stage III (n=10) | Stage IV (n=10) |
|---|---|---|---|---|---|
| **Race** | | | | | |
| White | 75 | 100 | 70 | 50 | 80 |
| Black | 17.5 | 0 | 20 | 50 | 0 |
| Latina | 2.5 | 0 | 10 | 0 | 0 |
| Multiracial | 5 | 0 | 0 | 0 | 20 |
| **Ethnicity** | | | | | |
| Non-Spanish, Non-Hispanic | 97.5 | 100 | 90 | 100 | 100 |
| Spanish; Hispanic | 2.5 | 0 | 10 | 0 | 0 |
| **Age** | | | | | |
| 30-39 | 10 | 0 | 20 | 0 | 20 |
| 40-49 | 22.5 | 0 | 20 | 30 | 40 |
| 50-59 | 17.5 | 10 | 20 | 30 | 10 |
| 60-69 | 17.5 | 20 | 10 | 10 | 30 |
| 70-79 | 27.5 | 50 | 30 | 30 | 0 |
| 80-89 | 5 | 20 | 0 | 0 | 0 |
| **Smoking History** | | | | | |
| Never | 60 | 60 | 60 | 40 | 80 |
| Previous | 32.5 | 30 | 40 | 40 | 20 |
| Current | 7.5 | 10 | 0 | 20 | 0 |
| **Tissue Site** | | | | | |
| Breast | 85 | 90 | 100 | 90 | 60 |
| Lymph Node | 2.5 | 10 | 0 | 0 | 0 |
| Brain | 7.5 | 0 | 0 | 10 | 20 |
| Ovary | 2.5 | 0 | 0 | 0 | 10 |
| Liver | 2.5 | 0 | 0 | 0 | 10 |
| **Prognosis** | | | | | |
| Primary | 82.5 | 90 | 10 | 90 | 50 |

| | | | | | |
|---|---|---|---|---|---|
| Recurrence | 5 | 10 | 0 | 0 | 10 |
| Metastatic | 12.5 | 0 | 0 | 10 | 40 |
| **Histology** | | | | | |
| Invasive | 2.5 | 0 | 0 | 0 | 0 |
| Invasive Ductal | 65 | 10 | 80 | 50 | 50 |
| Invasive Lobular | 2.5 | 80 | 0 | 10 | 0 |
| Invasive Mammary | 12.5 | 0 | 20 | 20 | 10 |
| Adenocarcinoma | 7.5 | 0 | 0 | 0 | 20 |
| Invasive Ductal Adenocarcinoma | 2.5 | 10 | 0 | 10 | 0 |
| Carcinoma | 2.5 | 0 | 0 | 0 | 10 |
| Lobular Carcinoma | 2.5 | 0 | 0 | 0 | 10 |
| Other | 5 | 0 | 0 | 20 | 0 |

**Table S2.** Lung cancer patient demographics

| Patient Characteristics (%) | Entire Population (n=40) | Stage I (n=10) | Stage II (n=10) | Stage III (n=10) | Stage IV (n=10) |
|---|---|---|---|---|---|
| **Sex** | | | | | |
| Male | 57.5 | 40 | 70 | 70 | 50 |
| Female | 42.5 | 60 | 30 | 30 | 50 |
| **Race** | | | | | |
| White | 75 | 10 | 70 | 80 | 70 |
| Black | 22.5 | 10 | 30 | 20 | 30 |
| Latinx | 2.5 | 80 | 0 | 0 | 0 |
| **Age** | | | | | |
| 40-49 | 5 | 10 | 0 | 10 | 0 |
| 50-59 | 15 | 30 | 10 | 0 | 20 |
| 60-69 | 57.5 | 50 | 50 | 70 | 60 |
| 70-79 | 22.5 | 10 | 40 | 20 | 20 |
| **Smoking History** | | | | | |
| Never | 7.5 | 0 | 0 | 10 | 20 |

| | | | | | |
|---|---|---|---|---|---|
| Previous | 55 | 70 | 70 | 70 | 10 |
| Current | 37.5 | 30 | 30 | 20 | 70 |
| **Prognosis** | | | | | |
| Primary | 92.5 | 90 | 100 | 100 | 80 |
| Metastatic | 7.5 | 10 | 0 | 0 | 20 |
| **Histology** | | | | | |
| Adenocarcinoma | 62.5 | 100 | 20 | 60 | 70 |
| Squamous Cell | 32.5 | 0 | 80 | 40 | 10 |
| Large Cell | 5 | 0 | 0 | 0 | 20 |

**Table S3.** Colon cancer patient demographics

| Patient Characteristics (%) | Entire Population (n=37) | Stage I (n=10) | Stage II (n=10) | Stage III (n=7) | Stage IV (n=10) |
|---|---|---|---|---|---|
| **Sex** | | | | | |
| Female | 48.7 | 80 | 60 | 71.4 | 50 |
| Male | 51.3 | 20 | 40 | 28.6 | 50 |
| **Age** | | | | | |
| 20-29 | 2.7 | 10 | 0 | 0 | 10 |
| 40-49 | 5.41 | 30 | 0 | 0 | 10 |
| 50-59 | 32.43 | 10 | 50 | 14.29 | 30 |
| 60-69 | 18.92 | 40 | 20 | 57.14 | 0 |
| 70-79 | 29.73 | 10 | 30 | 28.57 | 10 |
| 80-89 | 10.81 | 10 | 0 | 0 | 40 |
| **Race** | | | | | |
| White | 81.1 | 80 | 70 | 85.7 | 90 |
| Black, African American | 18.9 | 20 | 30 | 14.3 | 10 |
| **Smoking** | | | | | |
| Not documented | 8.1 | 10 | 0 | 28.5 | 0 |
| Never smoked | 45.9 | 40 | 50 | 42.9 | 50 |
| Previous smoker | 35.1 | 40 | 40 | 14.3 | 40 |
| Current smoker | 10.8 | 10 | 10 | 14.3 | 10 |
| **Prognosis** | | | | | |
| Primary | 64.9 | 100 | 40 | 85.7 | 40 |
| Recurrence | 5.4 | 0 | 0 | 0 | 20 |
| Metastatic | 29.7 | 0 | 60 | 14.3 | 40 |
| **Sample Type** | | | | | |
| EDTA Plasma | 54.1 | 50 | 50 | 71.4 | 50 |
| Serum | 45.9 | 50 | 50 | 28.6 | 50 |

**Table S4.** Galectin concentration values for patient groups in Figure 1

| Galectin | Group | N | Mean (ng/mL) | Std Dev | Range (ng/mL) | 5% Quantile | 95% Quantile | p-value |
|---|---|---|---|---|---|---|---|---|
| Gal-1 | Healthy | 36 | 18.1 | 3.40 | | | | |
| | Breast | 40 | 23.40 | 7.14 | 11.30 - 43.92 | 13.64 | 37.15 | 0.0007 |
| | Colon | 37 | 34.33 | 16.85 | 5.38 - 88.38 | 15.73 | 73.07 | ≤0.0001 |
| | Lung | 40 | 26.83 | 12.81 | 13.41 - 83.69 | 15.28 | 47.67 | 0.0002 |
| Gal-3 | Healthy | 36 | 6.73 | 2.00 | | | | |
| | Breast | 40 | 17.05 | 6.65 | 8.85 - 38.20 | 9.05 | 31.68 | ≤0.0001 |
| | Colon | 37 | 13.56 | 6.67 | 3.21 - 30.12 | 4.88 | 29.91 | ≤0.0001 |
| | Lung | 39 | 17.84 | 6.90 | 4.52 - 37.82 | 6.55 | 29.79 | ≤0.0001 |
| Gal-7 | Healthy | 27 | 2.12 | 5.15 | 0.15 - 27.26 | 0.16 | 18.66 | |
| | Breast | 40 | 1.70 | 1.75 | 0.46 - 11.69 | 0.54 | 3.34 | .0.497 |
| | Colon | 36 | 0.93 | 0.70 | 0.22 - 3.77 | 0.23 | 2.11 | |
| | Lung | 40 | 1.90 | 1.11 | 0.42 - 5.08 | 0.43 | 4.60 | 0.0071 |
| Gal-8 | Healthy | 15 | 2.00 | 1.73 | 0.46 - 5.97 | 0.46 | 5.97 | |
| | Breast | 34 | 2.42 | 2.21 | 0.95 - 11.84 | 0.95 | 7.63 | |
| | Colon | 28 | 2.48 | 3.16 | 0.58 - 13.19 | 0.60 | 12.30 | |
| | Lung | 32 | 2.67 | 7.89 | 0.56 - 45.71 | 0.57 | 18.82 | |
| Gal-9 | Healthy | 36 | 7 | 1.63 | | | | |
| | Breast | 40 | 8.44 | 3.06 | 3.42 - 16.12 | 4.26 | 14.60 | |
| | Colon | 37 | 9.92 | 3.89 | 5.27 - 25.12 | 5.42 | 18.47 | 0.0005 |
| | Lung | 40 | 10.12 | 4.81 | 3.79 - 26.64 | 4.92 | 25.73 | 0.0002 |

**Table S5.** Galectin concentration values for patient groups in Figure 2

| Gal | Cancer | Stage | Mean (ng/mL) | Std Dev | Range (ng/mL) | 5% Quantile | 95% Quantile | p-value |
|---|---|---|---|---|---|---|---|---|
| Gal-1 | Breast | I | 25.83 | 8.86 | 15.03 - 37.22 | 15.03 | 37.22 | 0.0456 |
| | | II | 20.47 | 5.22 | 11.30 - 28.36 | 11.30 | 28.36 | |
| | | III | 25.33 | 7.84 | 13.56 - 43.92 | 13.56 | 43.92 | 0.0025 |
| | | IV | 21.95 | 5.57 | 15.04 - 32.13 | 15.04 | 32.13 | |
| | Colon | I | 33.14 | 12.89 | 20.43 - 63.99 | 20.43 | 63.99 | ≤0.0001 |
| | | II | 35.80 | 22.33 | 5.38 - 88.38 | 5.38 | 88.38 | 0.0022 |
| | | III | 33.21 | 18.56 | 21.06 - 71.37 | 21.06 | 71.37 | 0.0026 |
| | | IV | 34.82 | 15.37 | 16.89 - 61.31 | 16.89 | 61.31 | 0.0014 |
| | Lung | I | 27.06 | 8.22 | 16.80 - 42.35 | 16.80 | 42.35 | 0.0036 |
| | | II | 32.28 | 21.31 | 16.39 - 83.69 | 16.39 | 83.69 | 0.0364 |
| | | III | 25.05 | 7.62 | 15.37 - 40.34 | 15.37 | 40.34 | 0.0179 |
| | | IV | 22.91 | 8.79 | 13.41 - 39.01 | 13.41 | 39.01 | |
| Gal-3 | Breast | I | 18.01 | 7.37 | 9.02 - 31.70 | 9.02 | 31.70 | ≤0.0001 |
| | | II | 17.77 | 4.44 | 11.50 - 23.98 | 11.50 | 23.98 | ≤0.0001 |

| | | | | | | | | |
|---|---|---|---|---|---|---|---|---|
| | | III | 16.86 | 8.74 | 8.85 - 38.20 | 8.85 | 38.20 | ≤0.0001 |
| | | IV | 15.58 | 6.08 | 9.69 - 31.35 | 9.69 | 31.35 | ≤0.0001 |
| | Colon | I | 14.64 | 6.92 | 8.09 - 29.89 | 8.09 | 29.89 | ≤0.0001 |
| | | II | 14.41 | 8.08 | 7.14 - 30.12 | 7.14 | 30.12 | 0.0002 |
| | | III | 13.53 | 6.87 | 3.21 - 24.74 | 3.21 | 24.74 | 0.0235 |
| | | IV | 11.65 | 5.23 | 5.07 - 20.51 | 5.07 | 20.51 | 0.0152 |
| | Lung | I | 19.19 | 6.46 | 11.14 - 29.79 | 11.14 | 29.79 | ≤0.0001 |
| | | II | 17.78 | 9.03 | 4.52 - 37.82 | 4.52 | 37.82 | 0.0005 |
| | | III | 16.23 | 4.93 | 9.09 - 24.68 | 9.09 | 24.68 | ≤0.0001 |
| | | IV | 18.18 | 7.35 | 6.55 - 28.08 | 6.55 | 28.08 | 0.0004 |
| Gal-7 | Breast | I | 2.10 | 0.80 | 1.00 - 3.37 | 1.00 | 3.37 | 0.0206 |
| | | II | 2.40 | 3.28 | 1.00 - 11.69 | 1.00 | 11.69 | |
| | | III | 1.11 | 0.39 | 0.58 - 1.95 | 0.58 | 1.95 | |
| | | IV | 1.18 | 0.49 | 0.46 - 1.69 | 0.46 | 1.69 | |
| | Colon | I | 0.82 | 0.57 | 0.22 - 1.68 | 0.22 | 1.68 | |
| | | II | 0.68 | 0.35 | 0.23 - 1.24 | 0.23 | 1.24 | |
| | | III | 1.38 | 1.23 | 0.23 - 3.77 | 0.23 | 3.77 | |
| | | IV | 0.96 | 0.48 | 0.41 - 1.73 | 0.41 | 1.73 | |
| | Lung | I | 1.15 | 0.67 | 0.42 - 2.58 | 0.42 | 2.58 | |
| | | II | 2.36 | 1.43 | 0.95 - 5.07 | 0.95 | 5.07 | 0.0341 |
| | | III | 2.09 | 1.18 | 0.99 - 4.35 | 0.99 | 4.35 | |
| | | IV | 1.99 | 0.76 | 1.05 - 3.20 | 1.05 | 3.20 | 0.0341 |
| Gal-8 | Breast | I | 2.41 | 1.82 | 0.95 - 6.22 | 0.95 | 6.22 | |
| | | II | 2.62 | 1.87 | 0.96 - 6.05 | 0.96 | 6.05 | |
| | | III | 3.02 | 3.49 | 0.95 - 11.84 | 0.95 | 11.84 | |
| | | IV | 1.64 | 0.89 | 1.02 - 3.77 | 1.02 | 3.77 | |
| | Colon | I | 3.12 | 3.78 | 0.67 - 11.21 | 0.67 | 11.21 | |
| | | II | 1.98 | 1.63 | 0.62 - 4.51 | 0.62 | 4.51 | |
| | | III | 3.14 | 4.99 | 0.58 - 13.19 | 0.58 | 13.19 | |
| | | IV | 1.65 | 1.72 | 0.74 - 5.13 | 0.74 | 5.13 | |
| | Lung | I | 1.61 | 1.22 | 0.57 - 4.35 | 0.57 | 4.35 | |
| | | II | 0.99 | 0.45 | 0.58 - 1.79 | 0.58 | 1.79 | |
| | | III | 1.15 | 0.50 | 0.56 - 1.80 | 0.56 | 1.80 | |
| | | IV | 6.85 | 15.71 | 0.70 - 45.71 | 0.70 | 45.71 | |
| Gal-9 | Breast | I | 9.31 | 2.62 | 5.76 - 12.54 | 5.76 | 12.54 | |
| | | II | 7.59 | 3.35 | 4.26 - 12.78 | 4.26 | 12.78 | |
| | | III | 8.39 | 3.21 | 3.42 - 16.12 | 3.42 | 16.12 | |
| | | IV | 8.48 | 3.24 | 4.78 - 14.69 | 4.78 | 14.69 | |
| | Colon | I | 9.04 | 2.53 | 5.79 - 12.00 | 5.79 | 12.00 | |
| | | II | 9.46 | 3.24 | 5.27 - 15.85 | 5.27 | 15.85 | |

| | | | | | | | |
|---|---|---|---|---|---|---|---|
| | III | 10.57 | 6.47 | 6.97 - 25.12 | 6.97 | 25.12 | |
| | IV | 10.81 | 3.65 | 5.44 - 17.73 | 5.44 | 17.73 | 0.0044 |
| Lung | I | 11.27 | 4.16 | 4.92 - 20.52 | 4.92 | 20.52 | 0.0030 |
| | II | 10.22 | 6.02 | 5.29 - 26.00 | 5.29 | 26.00 | |
| | III | 8.30 | 2.59 | 3.79 - 12.65 | 3.79 | 12.65 | |
| | IV | 10.69 | 5.86 | 6.61 - 26.64 | 6.61 | 26.64 | 0.0179 |
